# Tuning the Morphology of Immiscible Polymer Blend-Based Hybrid Nanocomposite for Improving Microwave Absorption Response

**DOI:** 10.1021/acspolymersau.4c00087

**Published:** 2024-11-28

**Authors:** Erick Gabriel Ribeiro dos Anjos, Tayra Rodrigues Brazil, Mirabel Cerqueira Rezende, Juliano Marini, Uttandaraman Sundararaj, Luiz Antonio Pessan, Fabio Roberto Passador

**Affiliations:** † Graduate Program in Materials Science and Engineering, Federal University of São Carlos (UFSCar), Rodovia Washington Luís, Km 235, São Carlos, São Paulo 13565-905, Brazil; ‡ Department of Science and Technology, Federal University of São Paulo (UNIFESP), 330 Talim St., São José dos Campos, São Paulo 12231-280, Brazil; § Department of Chemical and Petroleum Engineering, University of Calgary, 2500 University Drive NW, Calgary, Alberta T2N1N4, Canada; ∥ Department of Materials Engineering, Federal University of São Carlos (UFSCar), Rodovia Washington Luís, Km 235, São Carlos, São Paulo 13565-905, Brazil

**Keywords:** hybrid nanocomposites, polymer blends, electromagnetic compatibility, microwave absorption materials (MAM), reflection loss, electromagnetic behavior

## Abstract

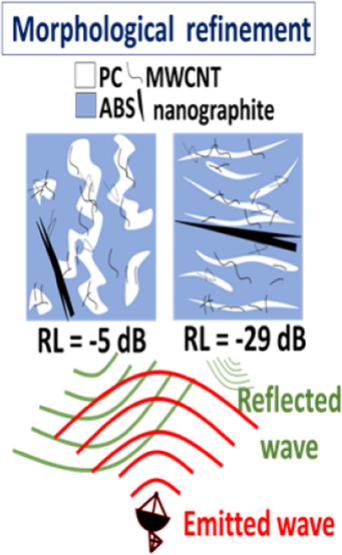

Polymer-blend-based nanocomposites incorporating carbon nanomaterials hold significant potential for microwave absorption materials (MAM) applications. This study investigates the microwave absorption response of hybrid nanocomposites composed of multiwalled carbon nanotubes (MWCNT) and nanographite, prepared using industrial-like melt-mixing masterbatch strategies in a polycarbonate/acrylonitrile-butadiene-styrene copolymer (PC/ABS) blend matrix with varying blend ratios (100/0, 80/20, 60/40, 50/50, 40/60, 20/80, and 0/100) and a constant filler content (2 wt % MWCNT and 2 wt % nanographite). Furthermore, the PC/ABS (40/60) blend-based nanocomposite was prepared with the addition of a compatibilizer, 5 wt % of maleic anhydride grafted ABS (ABS-*g*-MAH), to verify possible changes in morphology. Morphology, rheology, mechanical, electrical, and electromagnetic properties were correlated. From a morphological perspective, a preferential distribution of MWCNTs within the PC phase was observed, with the different blend ratios leading to a transition from a dispersed matrix morphology in 80/20 and 20/80 (PC/ABS) to cocontinuous morphologies in the intermediate blends (60/40, 50/50, and 40/60). The addition of ABS-*g*-MAH as a compatibilizer resulted in significant morphological refinement. Electromagnetic properties, evaluated using both X-band rectangular waveguide and broadband coaxial airline techniques, as well as electrical conductivity, were found to be strongly influenced by the varying morphologies. The nanocomposite PC/ABS/ABS-*g*-MAH with a thickness of 3.0 mm presented a Reflection Loss (RL) of −29.4 dB at 9.44 GHz, with a bandwidth of 3 GHz. Across the broadband spectrum, RL values below −10 dB were observed, including at lower frequencies around 3.70 GHz. These findings suggest that morphological tuning of the polymer matrix offers a promising pathway for optimizing microwave absorption in hybrid nanocomposites.

## 1 Introduction

Microwave Absorption Materials (MAMs) are critical in sectors such as telecommunications (e.g., antennas), medicine (e.g., medical devices), and the military (e.g., stealth technologies), where controlling microwaves and mitigating their adverse effects are paramount.^1−3^ Unlike electromagnetic shielding structures, MAMs are designed to minimize reflectivity, typically characterized by their Reflection Loss (RL) response, with an RL < −10 dB over a broader frequency range (bandwidth).^1−3^ Polymer nanocomposites are particularly promising for this purpose due to their lightweight nature, flexibility, ease of processing, and customizable mechanical and electromagnetic properties.^1^

Commonly used nanomaterials in these nanocomposites include metal oxides (primarily ferrites), multiwalled carbon nanotubes (MWCNT), and graphene-related materials (GRM).^1−3^ Among these, carbon *sp*^*2*^-hybridized nanomaterials like MWCNTs and graphene derivatives offer the advantage of maintaining the lightweight characteristics of polymer nanocomposites.^3 −5^ When used together in hybrid nanocomposites, these materials often exhibit a synergistic effect,^6−9^ enhancing the RL response compared to single-filled composites.^10,11^ Notably, hybrids of MWCNTs and GRM (as e.g., graphene nanoplatelets (GNP), graphene oxide (GO), and nanographite) have demonstrated superior performance in promoting good RL results.^10−12^ Nanographite, among GRM, offers promising electrical properties while being more cost-effective. This advantage arises from its production as a byproduct of graphene nanoplatelets, synthesized through the liquid exfoliation of natural graphite.^12,13^

The polymer matrix in nanocomposites also plays a crucial role, influencing the mechanical properties, processability, and thermal performance of the products. Recently, immiscible polymer blends have garnered attention as matrices for multifunctional nanocomposites.^14^ Phenomena such as the selective or preferential localization of nanomaterials enable the design of nanocomposite morphology at the microscopic level.^14,15^ Among the most widely used polymer blends in the automotive, telecommunications, and electronics industries are polycarbonate (PC) and acrylonitrile-butadiene-styrene copolymer (ABS), which combine the excellent mechanical and thermal properties of PC with the enhanced impact strength, processability, and cost-effectiveness of ABS.^16,17^

The selective distribution of nanomaterials within specific phases of a polymer blend is influenced by both thermodynamic behavior and kinetic factors, such as viscosity ratio, nanomaterial shape, and processing parameters (e.g., blending protocol, mixing time, and applied shear force).^14,18−20^ Previous works^21−26^ suggest that MWCNTs in PC/ABS blends tend to localize preferentially in the PC phase, with factors like the amount of polybutadiene (PB) in ABS^23,24^ and kinetic parameters such as blending protocol^27^ and polymer viscosities^18,19^ playing significant roles. The addition of compatibilizer agents may also be relevant for refining the morphology and controlling the fillers dispersion.^27−30^

In previous studies, our group evaluated the addition of MWCNTs^27^ and graphene nanoplatelets (GNPs)^31^ to PC/ABS blends, as well as the impact of different graphene-related materials on hybrid nanocomposites.^7,13^ For PC/ABS/MWCNT nanocomposites, we also assessed the effects of blending protocols and the addition of a compatibilizer agent (maleic anhydride grafted ABS—ABS-*g*-MAH), finding that a one-step extrusion process was the most effective for producing a conductive material with superior shielding performance.^27^ Specifically, in one study, we achieved commercial-grade attenuation levels of 20 dB in PC/ABS/MWCNT/GNP nanocomposites, demonstrating their potential as shielding materials.^13^

Building on these studies, this work investigates the effect of tuning matrix morphology in PC/ABS blend-based hybrid nanocomposites with the addition of MWCNT and nanographite. These materials were prepared using an industrial-like masterbatch dilution strategy through melt-mixing. The different PC/ABS morphologies obtained significantly impacted the electrical and electromagnetic properties, which were further tuned by refining the PC phase. Additionally, electromagnetic evaluations were conducted in the X-band as well as in a broadband frequency range, revealing that adjusting the blend ratio could be an effective approach for targeting different frequencies in this nanocomposite system while maintaining a consistent thickness. The RL behavior of the nanocomposites was evaluated both experimentally and through mathematical simulations.

## 2 Experimental Section

### 2.1 Materials

Two commercially available polymers were selected as the matrices: Polycarbonate (PC) grade LEXAN 123R, supplied by SABIC (Saudi Arabia), with a density of 1.20 g/cm^3^ and a high melt volume flow rate (MVR) of 21.0 cm^3^/10 min (300 °C/1.2 kg), and acrylonitrile-butadiene-styrene copolymer (ABS) (CYCOLAC Resin MG47) provided by SABIC Innovative Plastics South America (Brazil), with a density of 1.04 g/cm^3^ and a melt flow index (MFI) of 5 g/10 min (230 °C/3.8 kg).

The carbon nanomaterials used were also commercially sourced: Multiwalled carbon nanotubes (MWCNT) Nanocyl 7000, supplied by Nanocyl (Belgium), with an average diameter of 9.5 nm, an average length of 1.5 μm, 90% purity, and a surface area of 250–300 m^2^/g; and nanosized graphite (nanographite) provided by MGgrafeno (Brazil) featuring an average diameter of 30 nm (10 to 100 graphene layers), an average lateral size of 104 μm, and 99% purity.

For one composition of the nanocomposites, a compatibilizer agent was used: maleic anhydride grafted ABS (ABS-*g*-MAH) with 0.6 wt % of MAH. This compatibilizer agent was prepared on a laboratory scale via reactive extrusion. A complete description and characterization of this agent are detailed in dos Anjos (2020).^32^

### 2.2 Hybrid Nanocomposites Processing

The nanocomposites were prepared in two processing steps (masterbatch/dilution strategy). The masterbatch containing 10 wt % of each carbon nanomaterials (MWCNT and GMG) was prepared in the PC matrix, resulting in a total nanofiller content of 20 wt %. This masterbatch was then diluted in various PC/ABS blend ratios via extrusion to achieve a final composition with a constant 2 wt % of each nanomaterial (total 4 wt % of nanofillers). This percentage was selected based on a previous work using the same nanomaterials in a PC/ABS (85/15) blend.^13^ For comparison, hybrid nanocomposites with ABS and PC matrices were also prepared with 4 wt % nanofillers.

Prior to both extrusion steps, the materials were dried in a vacuum oven at 70 °C for 12 h. The extrusion process was carried out using a corotational twin-screw extruder (model AX16:40DR—AX Plásticos) with a screw diameter of 16 mm and an L/D ratio of 40. The screw profile included two kneading zones to ensure thorough mixing. The temperature profile was set as follows: 225/235/245/245/255 °C (from feed zone to die). The screw speeds were 50 rpm for the masterbatch preparation and 80 rpm for the dilution process, with feed screw speeds of 10 and 20 rpm, respectively. After extrusion, the nanocomposites were cooled in a 25 °C water bath and then pelletized. Table 1 provides an overview of the nanocomposite compositions, along with the nomenclature designated for each resulting material.

**Table 1:** Polymer Nanocomposite Compositions Nomenclature

Sample	PC (%)	ABS (%)	MWCNT (%)	nanographite (%)	ABS-*g-*MAH (%)
hPC	96.0	--	2	2	--
h80/20 (PC/ABS)	76.8	19.2	2	2	--
h60/40 (PC/ABS)	57.6	38.4	2	2	--
h50/50 (PC/ABS)	48.0	48.0	2	2	--
h40/60 (PC/ABS)	38.4	57.6	2	2	--
h20/80 (PC/ABS)	19.2	76.8	2	2	--
hABS	--	96.0	2	2	--
h40/60 (PC/ABS) + ABS-*g*-MAH	38.4	52.6	2	2	5

To further refine the morphology, a compatibilizer agent (ABS-*g*-MAH) was added to the h40/60 (PC/ABS) blend by substituting 5 wt % of the ABS with the compatibilizer. This concentration of compatibilizing agent was selected based on previous works.^27,32^

Postextrusion, the nanocomposite pellets were dried again in a vacuum oven at 70 °C for 12 h. Rectangular samples for electrical conductivity, electromagnetic characterization, and Izod impact testing were prepared via injection molding using a lab-scale vertical injection molding machine (MH Equipamentos, Brazil). The injection molding parameters included a barrel temperature of 280 °C, a mold temperature of 70 °C, and an injection pressure of 8 atm. The mold dimensions were 60 mm × 12 mm × 3 mm, with subsequent adjustments made to fit the specific analyses.

Additionally, rectangular, coaxial, and tensile test specimens were prepared using a Carver hot press (Carver Inc., Wabash, IN, USA). The processing conditions for the hot press included a constant pressure of 7 atm, a temperature of 280 °C, and cooling with room-temperature water for a total time of 10 min.

### 2.3 Hybrid Nanocomposites Characterization

#### 2.3.1 Morphology

The morphology of the nanocomposites was examined using a TESCAN MIRA3 field-emission gun scanning electron microscope (FEG-SEM), operating at an acceleration voltage of 5 kV. Injection-molded samples were notched, cryo-fractured, and coated with a thin gold layer via sputtering. For samples with higher ABS content, additional cryo-fractured specimens were subjected to a superficial chemical etching treatment using a 30% (weight/volume) NaOH solution at 90 °C for 10 min.^33^ This treatment selectively removed the PC phase through depolymerization, allowing for clearer observation of the blend morphology.^33^

#### 2.3.2 Rheology

The viscosity of the neat polymers (PC and ABS) was evaluated under steady-state conditions using a Dynisco Galaxy V Capillary Rheometer. The measurements were performed at shear rates ranging from 2 to 2000 s^-1^, with a capillary diameter of 1.25 mm, an L/D ratio of 20, and a barrel temperature of 280 °C, following ASTM D3835–16. The Weissenberg-Rabinowitsch correction was applied to the data.

For the hybrid nanocomposites, rheological behavior was analyzed using Small-Amplitude Oscillatory Shear (SAOS) tests on a TA Instruments ARG2 stress-controlled rotational rheometer. The instrument was equipped with parallel-plate geometry (25 mm diameter and 1 mm gap). The analyses were conducted under a nitrogen atmosphere at a temperature of 280 °C. Prior to each test, strain sweep tests were performed to confirm that the applied strain amplitude of 0.5% was within the linear viscoelastic range.

#### 2.3.3 Impedance Spectroscopy (IS)

AC electrical conductivity was determined from impedance measurements obtained via impedance spectroscopy (IS). The analyses were conducted on injection-molded nanocomposite samples over a frequency range of 1 Hz to 1 MHz, with a voltage amplitude of 0.5 V at room temperature. The measurements were performed using a Solartron SI 1260 impedance analyzer.

#### 2.3.4 DC Electrical Conductivity

DC electrical conductivity was measured using a four-point probe setup with a Loresta GP conductivity meter, MCP-T610 model. A voltage of 90 V was applied to rectangular specimens prepared by compression molding to assess electrical conductivity.

#### 2.3.5 Electromagnetic Behavior (Shielding, Complex Properties, and Reflection Loss)

##### 2.3.5.1 Rectangular Waveguide (X-Band)

The electromagnetic behavior in the X-band frequency range (8.2–12.4 GHz) was evaluated using a vector network analyzer (VNA, Agilent Technologies, model PNA-L N5235A) coupled with a rectangular waveguide (WR-90), in accordance with ASTM D7449M-22a.^34^ The 3.0 mm thicker samples were adjusted to fit tightly inside the waveguide offset.

##### 2.3.5.2 Broadband (0.3 to 18 GHz)

Broadband analyses were conducted using a coaxial setup with a 7.0 mm 50 Ohm airline (85051BR03-Keysight) and calibration kit (85050D-Keysight) in an Agilent E5071C network analyzer (ENA series 300 kHz–20 GHz), following ASTM D5568–22a.^35^ Coaxial samples were compression-molded and adjusted to fit tightly inside the airline.

##### 2.3.5.3 Data Evaluation

For both analysis setups, the analyses were initially performed in transmission mode to obtain the complex scattering parameters (S_11_, S_21_, S_12_, and S_22_). From this data, the relative permittivity (ε_r_) and relative permeability (μ_r_) were calculated using the Nicolson-Ross-Weir (NRW) algorithm.^34,35^ The complex parameters were also used to calculate the transmission (T), reflection (R), and absorption (A) coefficients, as well as the shielding efficiency parameters: total shielding efficiency (SE_T_), shielding by reflection (SE_R_), and shielding by absorption (SE_A_), following the eqs 1–6, respectively.^36,37^
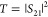
1
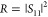
2
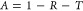
3
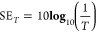
4
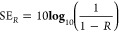
5
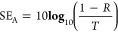
6

The reflection loss (RL) response of the samples was optimized by adjusting the thickness with sandpaper for the X-band and using the same thickness for coaxial analysis. For both setups, measurements were conducted in reflectance mode only, with a short circuit placed at the end of the waveguide or airline behind the sample to ensure a near-100% reflective surface.

#### 2.3.6 Mathematical Estimation of the RL

The RL was mathematically estimated for different thicknesses based on impedance matching phenomena from transmission line theory,^38,39^ which correlate the materials’ complex dielectric properties (μ_r_ and ε_r_), thickness, and electromagnetic wave characteristics, as calculated using eqs 7 and 8:
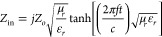
7
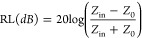
8

where *Z*_in_ represents the input impedance of the material, *Z*_0_ the impedance of the empty line (air, 50 Ω for both setups), *t* the layer thicknesses, *f* the electromagnetic frequency, and *c* the speed of light (3.0 × 10^8^ m/s).

#### 2.3.7 Tensile Tests

Compression-molded specimens, following the V-type shape of ASTM D638–06,^40^ were tested using an Instron machine (model 5965) with a load cell capacity of 5 kN, a crosshead speed of 5 mm/min, and at room temperature. Five specimens of each composition were tested.

#### 2.3.8 Izod Impact Strength

Izod impact strength tests were performed on notched injection-molded samples using a CEAST/Instron Izod impactor test machine (model 9050) following ASTM D256–18.^41^ The equipment was fitted with a 1 J hammer, and the specimens were notched using a manual notching machine (CEAST/Instron, model 9050) with 0.1-in. notches.

## 3 Results and Discussion

### 3.1 Morphological and Rheological Evaluation

**Figure 1:**
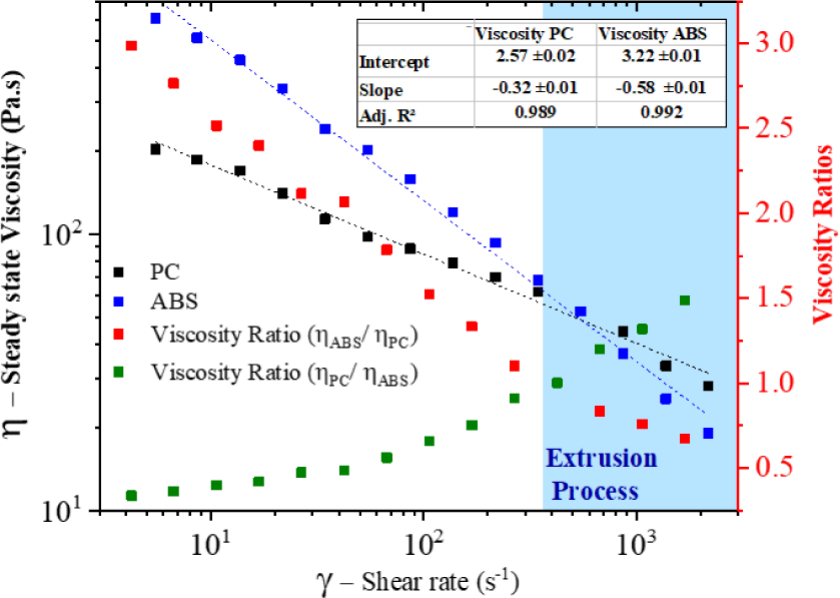
Viscosity versus shear rate curves obtained by capillary rheometry for neat PC and ABS.

In the development of polymer-blend nanocomposites for Material Microwave Absorbing Materials (MAMs) applications, a comprehensive understanding of morphology is crucial, as it directly the influences electromagnetic performance.^11^ Microrheology is a field in materials science that focuses on controlling and predicting the morphology of immiscible polymer blends,^42^ with viscosity correlation between the different phases being an important parameter for anticipating the resulting morphology.^42−45^ This analysis involved high shear rate viscosity data obtained through capillary rheometry and evaluating the viscosity ratio between the two polymers used in the polymer blends. Figure 1 shows the viscosity versus shear rate curves obtained by capillary rheometry for neat PC and ABS, along with the viscosity ratio.

Figure 1 shows that both polymers exhibit shear-thinning behavior across the studied shear rates, which can be represented by the Power Law model as a linear relationship. At lower shear rates, ABS displays higher viscosity values than PC. This difference can be attributed to the distinct structural characteristics of the polymers.

The viscosity ratios between the ABS and PC phases were observed to range from 1.0 to 0.6 at shear rates close to those found in extrusion processes (300–3000 s^-1^), which suggests the potential formation of fibrils, as described in Taylor’s studies on emulsions of Newtonian fluids.^42,46,47^ These fibrils may randomly break due to capillary instability mechanisms, leading to the formation of a dispersed phase, particularly in the PC-dominant blend. For the ABS-dominant phase blend (where ABS is more abundant in the composition), the viscosity ratios between the PC and ABS phases ranged from 1.6 to 1.0 at the same shear rate interval, indicating a possible coarser morphology with larger spherical-like PC domains.

**Figure 2:**
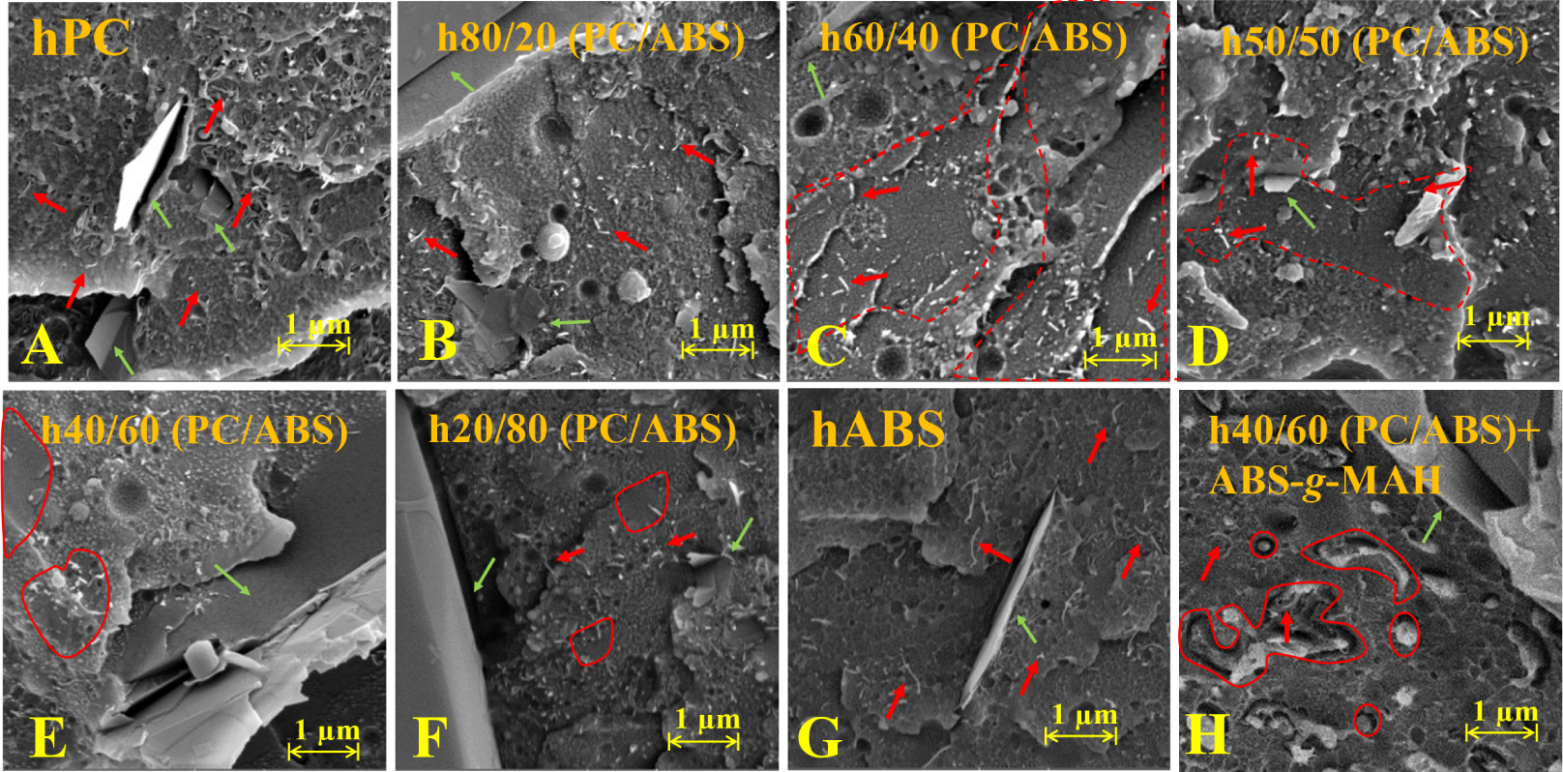
FEG-SEM micrographs of the PC/ABS blend-based hybrid nanocomposites with different blend ratios: (A) hPC, (B) h80/20 (PC/ABS), (C) h60/40 (PC/ABS), (D) h50/50 (PC/ABS), (E) h40/60 (PC/ABS), (F) h20/80 (PC/ABS), (G) hABS, and (H) h40/60 (PC/ABS)+ABS*-g*-MAH.

However, the formation of this dispersed phase in a polymer blend system, prepared by the complex flow of twin-screw extruder and later injection molding, is more complex. The associated mechanisms are also dependent on other parameters such as the blend ratio,^42^ interfacial tension,^42^ processing parameters, and, most importantly, the presence of fillers and/or a compatibilizer agent,^48^ such as ABS-*g*-MAH.^32^ All these factors were considered when further evaluated the morphology of the hybrid nanocomposites using FEG-SEM, as shown in Figure 2.

The surface morphology of hPC (Figure 2A) appeared more uniform and stretched compared to hABS (Figure 2G), where the heterogeneous structure of ABS resulted in a rougher surface with small spherical cavities corresponding to the PB domains. In both nanocomposites, the CNTs (indicated by red arrows) were well distributed throughout the polymer matrices.

As anticipated, PC/ABS is an immiscible polymer blend,^23,24^ and the matrix morphology was predominantly influenced by the blend ratio. A dispersed phase morphology was observed in blends with extreme ratios, where ABS domains were dispersed in a PC matrix for the 80/20 blend ratio, and conversely, PC domains were dispersed in an ABS matrix for the 20/80 blend ratio. In contrast, the intermediate blend ratios (60/40, 50/50, and 40/60) exhibited a cocontinuous morphology, reflecting the balance between the two polymer phases.

For the PC/ABS blend-based hybrid nanocomposites, the presence of both nanomaterials, MWCNTs and GMG (indicated by green arrows), was confirmed across all samples. However, evidence of a preferential localization of MWCNTs in the polymer blends was noted, consistent with findings in the literature.^21−26^ The MWCNTs appeared as shorter fragments and were often absent from specific areas of the matrix that exhibited smoother surfaces, likely corresponding to the PC phase. In contrast, the GMG showed a wide range of thicknesses and lateral sizes, often larger than the domains of the polymer blend phases, making it difficult to determine any clear preferential distribution pattern. Interestingly, in some compositions (hPC, h40/60 (PC/ABS), h20/80 (PC/ABS), hABS, and h40/60 (PC/ABS)+ABS-*g*-MAH), MWCNTs were observed in proximity to nanographite nanoplatelets, likely due to the high chemical affinity between the two nanofillers, driven by π–π chemical orbital interactions.^6,10^

**Figure 3:**
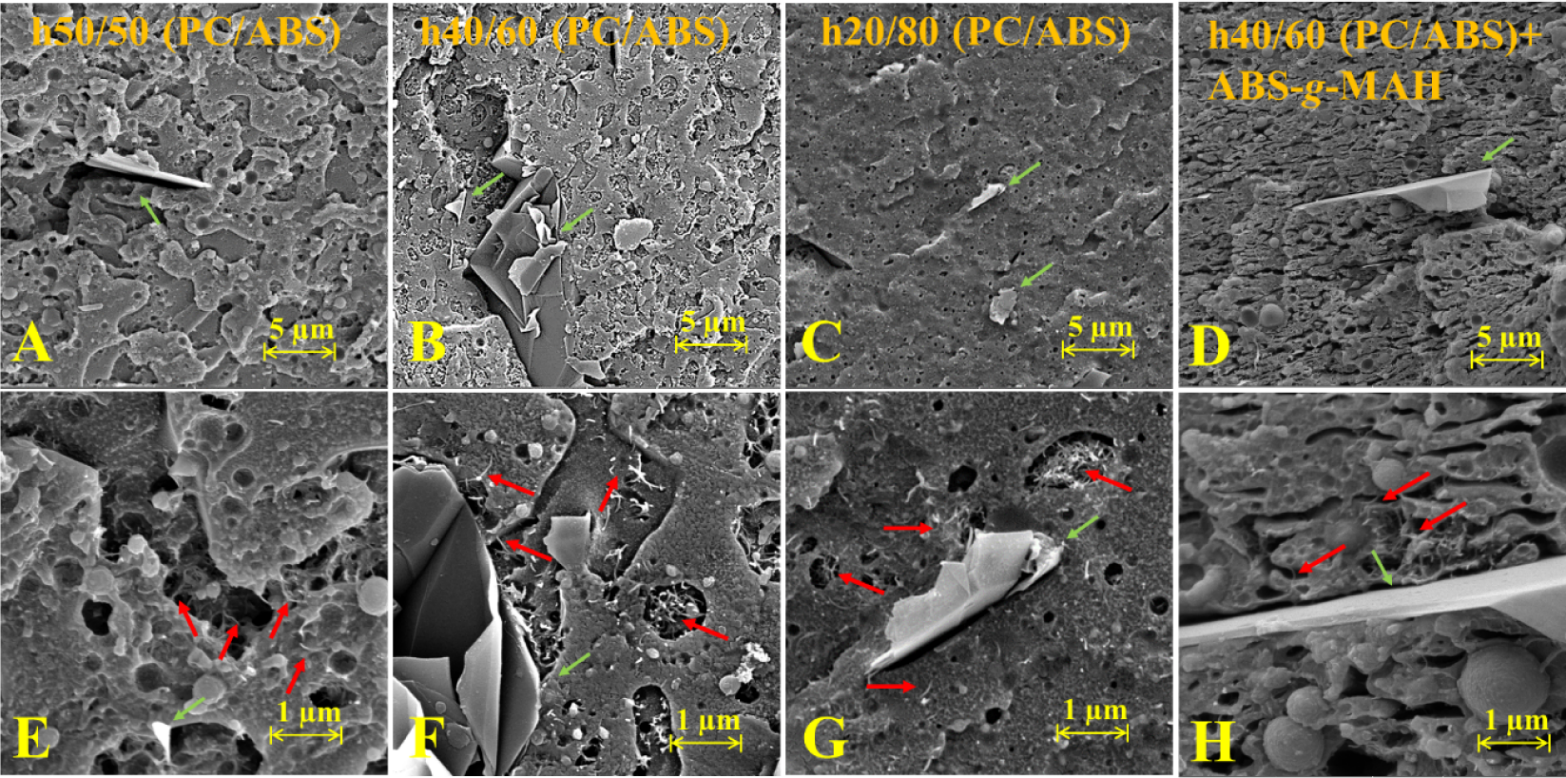
FEG-SEM micrographs of the PC/ABS blend-based hybrid nanocomposites with different blend ratios with PC phase extracted: (A and E) h50/50 (PC/ABS), (B and F) h40/60 (PC/ABS), (C and G) h20/80 (PC/ABS), and (D and H) h40/60 (PC/ABS)+ABS*-g*-MAH.

In the h80/20 (PC/ABS) nanocomposite, large spherical domains were observed, with some appearing to have been pulled out from the matrix, likely representing ABS domains. For other blend ratios, the PC domains were more distinct (areas outlined by red dashed lines). In the h20/80 (PC/ABS) blend, these domains appeared as dispersed phases within an ABS matrix. In contrast, in the h60/40, h50/50, and h40/60 (PC/ABS) nanocomposites exhibited cocontinuous morphologies. The addition of ABS-*g*-MAH in the h40/60 (PC/ABS) composition led to a refinement of the PC domains, and the resulting morphology (Figure 2H) closely resembled the one obtained by Jin et al.^49^ for ABS/PC blends (55/45) compatibilized with poly (methyl-methacrylate) (PMMA). To confirm these observations, further analysis was performed by selectively extracting PC from the surface of compositions with a higher ABS content (Figure 3).

From the PC phase extraction, the hypothesis of a cocontinuous matrix morphology was reinforced for the 50/50 and 40/60 (PC/ABS) blend ratios in the h50/50 (PC/ABS) and h40/60 (PC/ABS) compositions. In contrast, the polymer matrix of the h80/20 (PC/ABS) composition exhibited a distinct dispersed morphology with well-defined PC domains. Furthermore, in the h40/60 (PC/ABS) + ABS-*g*-MAH, the extraction of the PC phase revealed a cocontinuous morphology with enhanced phase interactions. This to a more homogeneous and thinner continuous PC phase compared to the noncompatibilized blends.

Upon evaluating the preferential localization of MWCNTs within the polymer blend matrices, distinct patterns emerged. In the h50/50 (PC/ABS) nanocomposite, most MWCNTs were removed along with the PC phase during the extraction process, with only a few remaining in the ABS matrix and some MWCNTs observed near the interfaces. For the h40/60 (PC/ABS) composition, MWCNT aggregates were found where the PC phase was removed, remaining entangled at the interfaces. Notably, these MWCNTs were longer compared to the tips observed before extraction, further reinforcing their preferential localization within the PC phase.

Similarly, in the h20/80 (PC/ABS) composition, MWCNT aggregates were observed in areas where PC domains were expected, with a higher migration of MWCNTs into the ABS matrix. The migration for the nonpreferred ABS phase has likely occurred due to the lower PC content and smaller size of the PC domains for this blend ratio. The morphologies observed after PC phase extraction strongly suggest that MWCNTs are preferentially localized in the PC phase and at the PC/ABS interfaces, confirming the expected behavior based on the polymer system and processing strategy used.

**Figure 4:**
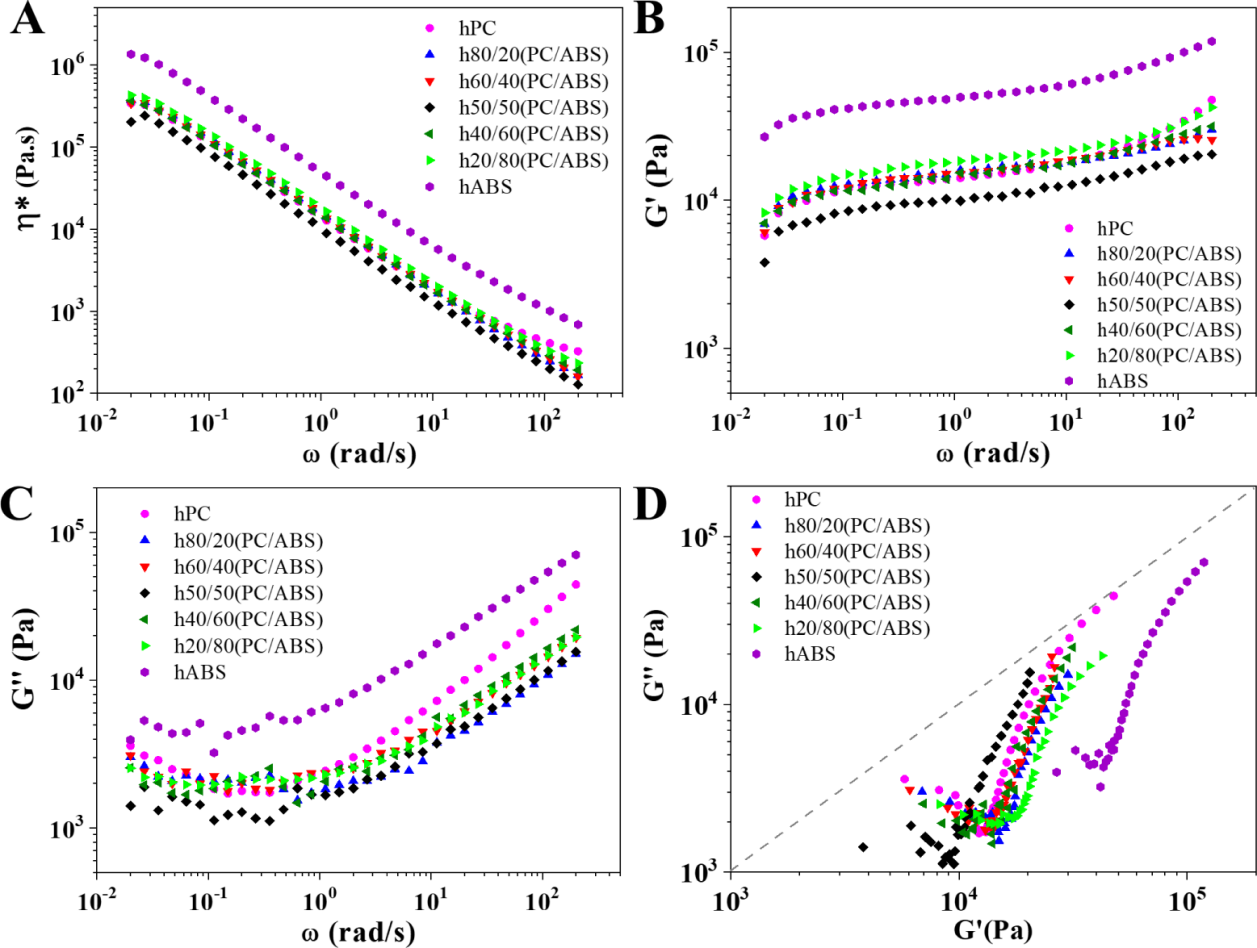
Rheological SAOS characterization of PC/ABS blend-based hybrid nanocomposites with different blend ratios: (A) Complex viscosity, (B) Storage moduli – *G*′, (C) Loss moduli - *G*″, and (D) Cole–Cole Plot.

Further insights into the interactions between nanomaterials and the polymer matrix were obtained using Small-Amplitude Oscillatory Shear (SAOS) rheological behavior, which provided additional details about the nanocomposites morphology^50^ (Figure 4). Among all compositions, the hABS nanocomposite exhibited the highest complex viscosity and shear modulus components. In contrast, the other polymer blends, including hPC, showed comparable rheological behavior. This difference can be attributed to the distinct architecture of ABS polymer chains, as previously discussed, particularly at lower shear rates in steady-state morphology.

The complex rheological properties of the nanocomposites did not show significant variations across different blend ratios, as observed in Figure 4A. Upon examining the storage modulus (*G*′) and loss modulus (*G*″), it was noted that the similar rheological behavior across the nanocomposites could be attributed to the consistent MWCNT and GMG content in all samples, which had a greater influence on rheological behavior. The nearly constant *G*′ values at lower angular frequencies for all nanocomposites, as observed in Figure 4B, along with the shift of values into the solid-like region on the Cole–Cole plot (Figure 4D), strongly suggest the formation of a nanofiller network.^50,51^

### 3.2 AC and DC Electrical Conductivity Behavior

**Figure 5:**
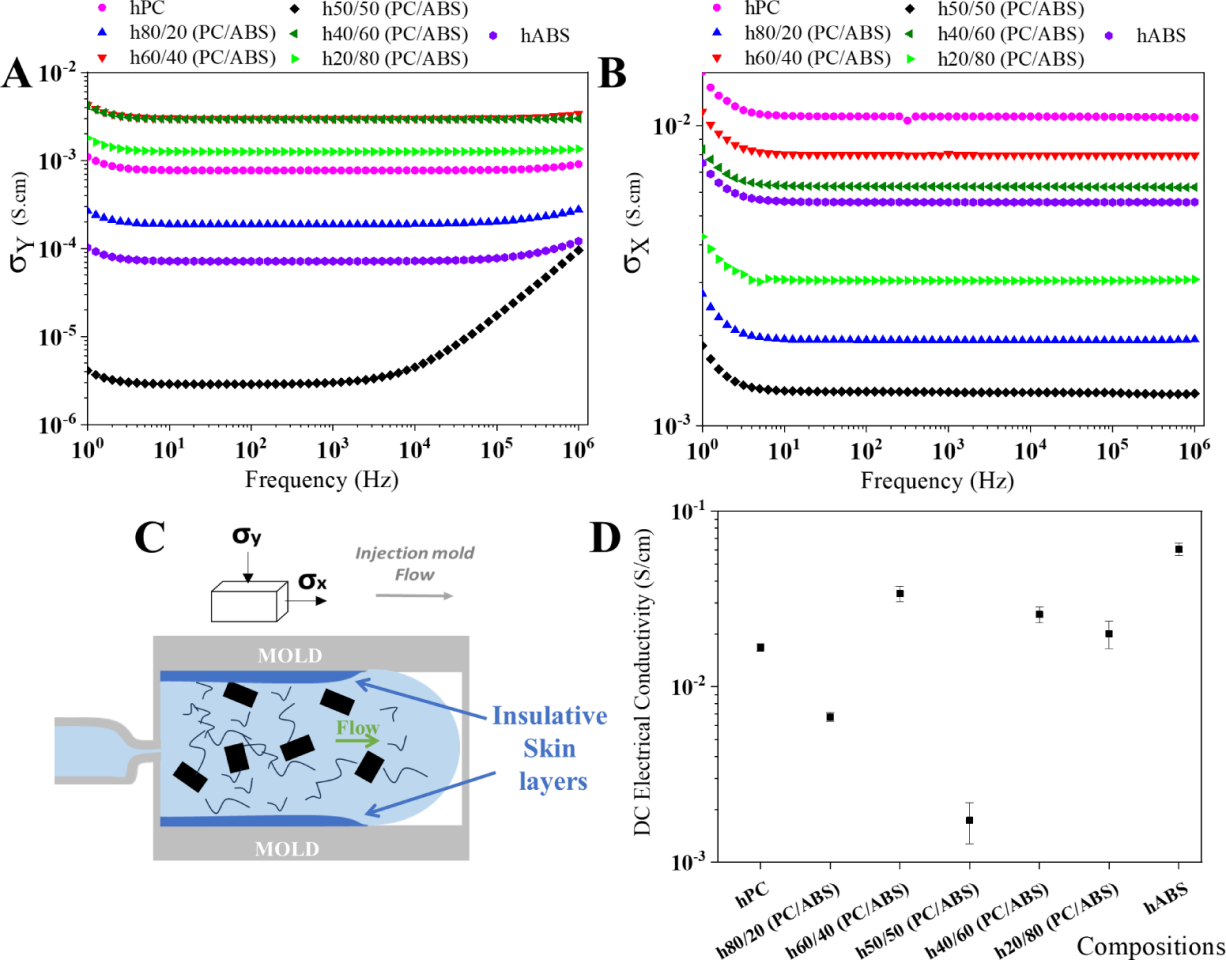
(A and B) AC electrical conductivity in different directions of the injection flow for injection-molded samples obtained by impedance spectroscopy, (C) representation of the anisotropy of the electrical behavior, and (D) DC electrical conductivity for hot-compressed samples obtained by the 4-probes method.

Electrical conductivity is intrinsically linked to the morphological characteristics of nanocomposites.^51^ In hybrid nanocomposites, it is anticipated that a three-dimensional conductive network composed of MWCNTs and GMG will develop within the polymer matrices.^6^ For immiscible polymer blend-based nanocomposites, where the nanofiller (MWCNT) shows preferential localization, conductivity can be enhanced by the formation of a double-percolated structure.^52,53^ This occurs when the nanofillers are distributed in a continuous phase with limited volume, a feature characteristic of cocontinuous morphologies.^52^ The electrical conductivity of these hybrid nanocomposites was evaluated using alternating current (AC) impedance spectroscopy for injection-molded samples and direct current (DC) four-point probe measurements for compression-molded samples (Figure 5). For the injection-molded samples, the orientation of the nanomaterials can affect the electrical behavior. Therefore, electrical properties were measured both in the direction of the injection flow (σ_*x*_) and perpendicular (σ_*y*_) to it.

The AC behavior of injection-molded samples revealed a highly conductive nature across all nanocomposites in the parallel injection mold-flow direction, with electrical conductivity values remaining consistent regardless of the electrical field oscillation frequency (Figure 5A). In contrast, the electrical conductivity values in the perpendicular injection flow direction varied significantly (Figure 5B). For example, compositions such as hABS and h50/50 (PC/ABS) exhibited electrical conductivity values several orders of magnitude lower than those observed in the parallel direction, with values of approximately 0.005 and 0.0015 S/cm in the parallel direction and 0.0001 and 3 × 10^–6^ S/cm in the perpendicular direction for hABS and h50/50 (PC/ABS), respectively, measured at 10 Hz. This anisotropic behavior aligns with findings in the literature regarding the injection mold skin effect.^10,25^ The high shear rates near the mold walls negatively impact the formation of conductive filler networks in the perpendicular direction, potentially creating an insulating skin and disrupting percolative pathways, as illustrated in Figure 5C.

For both measurement directions, the nanocomposites with PC/ABS blend ratios of 60/40 and 40/60 exhibited higher electrical conductivity values, suggesting the formation of a 3D-network of the nanofillers. In contrast, the nanocomposite with a 50/50 (PC/ABS) blend ratio showed the lowest electrical conductivity values in both directions. This reduced conductivity may be attributed to the equal content of both polymers, resulting in larger, more insulating ABS phases interspersed among the PC domains. Additionally, the more insulating ABS domains may preferentially form the insulating skin due to their lower viscosity under high shear rates during the injection molding process, which exceeds the shear rates measured in Figure 1.

The DC electrical conductivity of compression-molded samples exhibited a similar trend but with slightly higher values compared to the AC measurements in the parallel injection mold flow direction (Figure 5A). In compression molding, the shear rates applied are lower than the injection molding process, preserving the conductive nanofiller pathways and results in more homogeneous behavior. Notably, the DC electrical conductivity values for hPC and hABS differed, with compression-molded hABS showing higher DC electrical conductivity of 0.8 S/cm for the compressed sample, and AC electrical conductivity of 0.005 S/cm measured at 10 Hz in the parallel flow direction for hABS injection-molded samples. Despite this variation, all values are comparable and indicate that these composites exhibit high electrical conductivity (∼10^–3^ to 10^–2^ S/cm), making them suitable for electromagnetic interference (EMI) shielding applications and potentially beneficial for electrostatic discharge (ESD) protection.^10^

### 3.3 Effect of ABS-*G*-MAH on the Rheological and Electrical Behavior

**Figure 6:**
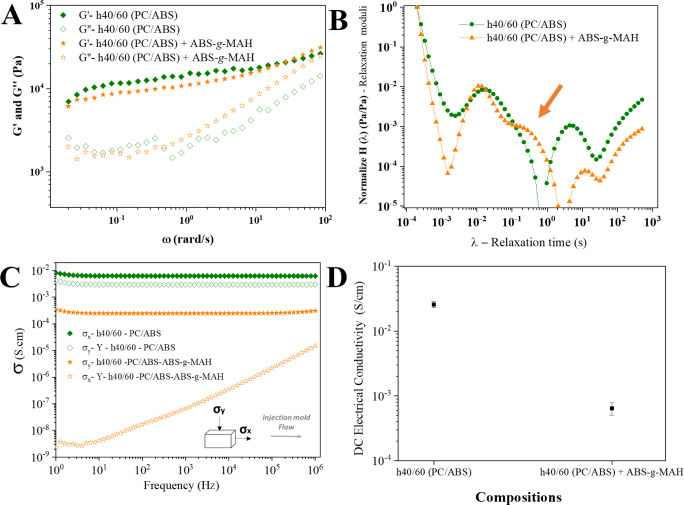
General comparison between the composition with and without the compatibilizer agent (ABS-*g*-MAH) the SAOS rheological behavior (A) *G*′ and *G*″, (B) relaxation moduli -H(λ), (C) AC electrical conductivity measured by impedance spectroscopy in different directions, and (D) DC electrical conductivity.

The impact of ABS-*g*-MAH addition was evident in the morphological changes observed in the polymer matrix, particularly in the refinement of PC domain sizes, as shown in Figures 2 and 3. This section focuses on the influence of this compatibilizer agent on the rheological and electrical properties of the nanocomposite (Figure 6).

In the rheological analysis, the complex shear moduli components were nearly consistent across the samples, with slightly higher *G*′ values observed for the h40/60 (PC/ABS) nanocomposite. Additionally, more continuous *G*″ values were noted for the h40/60 (PC/ABS) + ABS-*g*-MAH composition (Figure 6A). To further elucidate the differences in rheological behavior, the complex rheological properties were analyzed using the relaxation moduli H (λ) derived from the shear moduli data (Figure 6B).

The data on relaxation moduli (H(λ)) highlighted significant rheological changes in the materials, with multiple relaxation phenomena observed in the polymer blend-based nanocomposites. Phenomena with very short relaxation times (λ) are typically associated with the relaxation of polymer chains within the domains, whereas phenomena appearing at longer λ are linked to polymer chains near the blend interfaces or nanofiller interfaces, where mobility is highly restricted.^54,55^ The addition of ABS-*g*-MAH introduced a shoulder toward higher relaxation times in the peak originally centered at λ = 0.002 s for h40/60 (PC/ABS). This shift can be attributed to the reduced mobility of grafted chains within the ABS domains and the formation of a more restrictive interface, possibly due to the formation of some ABS-*co*-PC copolymer during processing.

The impact of ABS-*g*-MAH on electrical conductivity was substantial, significantly reducing conductivity in both AC (Figure 6C) and DC (Figure 6D) regimes. For AC measurements, the electrical conductivity of injection-molded samples with the compatibilizer agent decreased by a factor of 18 in the flow direction and more than 1000 times in the perpendicular direction, exhibiting behavior closer to an insulative material. This amplification of anisotropic behavior can be explained by the morphological refinement of PC domains in the matrix, where the 3D network becomes more susceptible to pathway breakage. The MWCNTs, preferentially dispersed in the thinner PC domains and PC-ABS interfaces, result in fewer contact points between MWCNTs and MWCNT-GMG. Consequently, the network’s susceptibility to breakage due to the addition of ABS-*g*-MAH also led to lower DC electrical conductivity in compression-molded samples (Figure 6D). These results align with the morphological observations and provide valuable insights into the electromagnetic behavior of these hybrid nanocomposites.

### 3.4 Electromagnetic Behavior of the Hybrids on the X-Band

**Figure 7:**
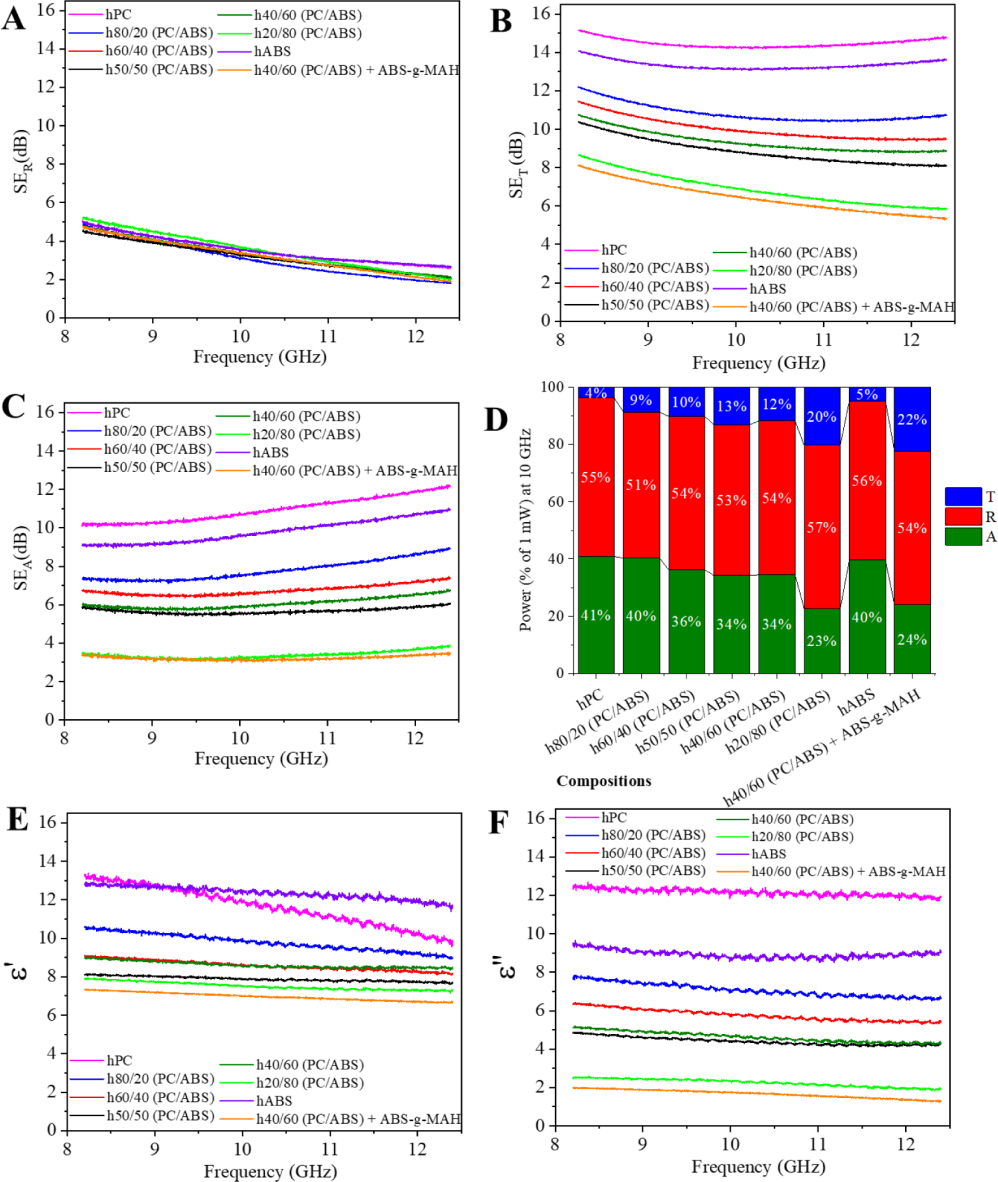
Electromagnetic shielding behavior in the X-band measured by rectangular waveguide – WR90 of the PC/ABS blend-based hybrid nanocomposites with different blend ratios: (A) attenuation by absorption -SE_A_, (B) attenuation by reflection -SE_R_, (C) total attenuation -SE_T_, and (D) power coefficients distribution at 10 GHz. Complex permittivity components: (E) real permittivity - ε’, (F) imaginary permittivity - ε″, obtained by NRW algorithm.

The electromagnetic behavior of nanocomposites is influenced not only by the type and content of conductive nanomaterials but also by the morphology of the matrix. As illustrated in Figure 7, polymer blend-based nanocomposites exhibited lower shielding effectiveness compared to hPC and hABS (single polymer matrix nanocomposites). The compositions hPC and hABS achieved total attenuations of approximately 14 and 16 dB, respectively, while all PC/ABS blend-based hybrid nanocomposites with different blend ratios exhibited attenuations below 12 dB.

The reflection component of shielding was similar across all nanocomposites, attributed to the uniform nanofiller content and electrical conductivity.^7^ However, differences in total attenuation behavior were primarily due to variations in the absorption shielding component. In these hybrid nanocomposites, the absorption mechanisms are mainly driven by interfacial polarization and ohmic losses, which arise from the 3D network of carbon nanomaterials. These mechanisms are influenced by the spatial arrangement of the nanofillers in the matrix—specifically, the proximity of conductive nanofillers (interfacial polarization) and the length of the conductive pathways (ohmic loss).^11^ In other words, morphology plays a crucial role. A sparser morphology, where MWCNTs are preferentially located in one phase, may result in shorter networks with fewer contact points, leading to a significant reduction in the absorption component for PC/ABS blend-based hybrid nanocomposites compared to ABS or PC nanocomposites.

This effect of a sparser, shorter network with fewer contact points is evident in the performance of the h20/80 (PC/ABS) and h40/60 (PC/ABS) + ABS-*g*-MAH nanocomposites. In h20/80 (PC/ABS), a significant amount of the MWCNTs is confined to the PC dispersed phase domain, while in h40/60 (PC/ABS) + ABS-*g*-MAH, the refined PC domains exhibit a cocontinuous morphology. Both compositions show the lowest shielding efficiency among the nanocomposites studied. These observations are corroborated by the power coefficients (A, R, and T) (Figure 7D), where these two compositions exhibited lower A coefficients (23% and 24%, respectively), indicating a higher amount of power transfer. Overall, even single-polymer matrix nanocomposites did not achieve the commercially required attenuation values, which generally require a minimum of 20 dB.^11^

The transmission analyses also included evaluations of complex electromagnetic properties, such as permittivity and permeability, using the NRW algorithm,^35^ presented in Figure 7E,F, respectively. Nanocomposites containing *sp*^*2*^-hybridized carbon nanomaterials primarily exhibit changes in permittivity, as these materials are conductive and do not significantly alter magnetic permeability, typically resulting in permeability values close to that of air (μ’ ∼ 1 and μ″ ∼ 0) (Figure S1).^7,11^ However, permittivity values are influenced by the content and distribution of these nanomaterials within the matrix, as shown in Figure 7E,F.

The real component of permittivity (ε’) is related to the in-phase electrical response and energy storage, primarily associated with reflection shielding phenomena. Conversely, the imaginary component of permittivity (ε’’) is linked to energy loss and absorption shielding phenomena.^1^ Consequently, the attenuation of electromagnetic waves propagating through a material is sometimes evaluated using the loss tangent, which is the ratio of ε’’ to ε’.^1^ For the materials under evaluation, single-polymer matrix nanocomposites (hPC and hABS) exhibited the highest values for both permittivity components. In contrast, the PC/ABS blend-based nanocomposites displayed slightly lower ε’ values and a varied range of ε’’ values, likely due to different morphologies that do not favor dissipative loss phenomena.

**Figure 8:**
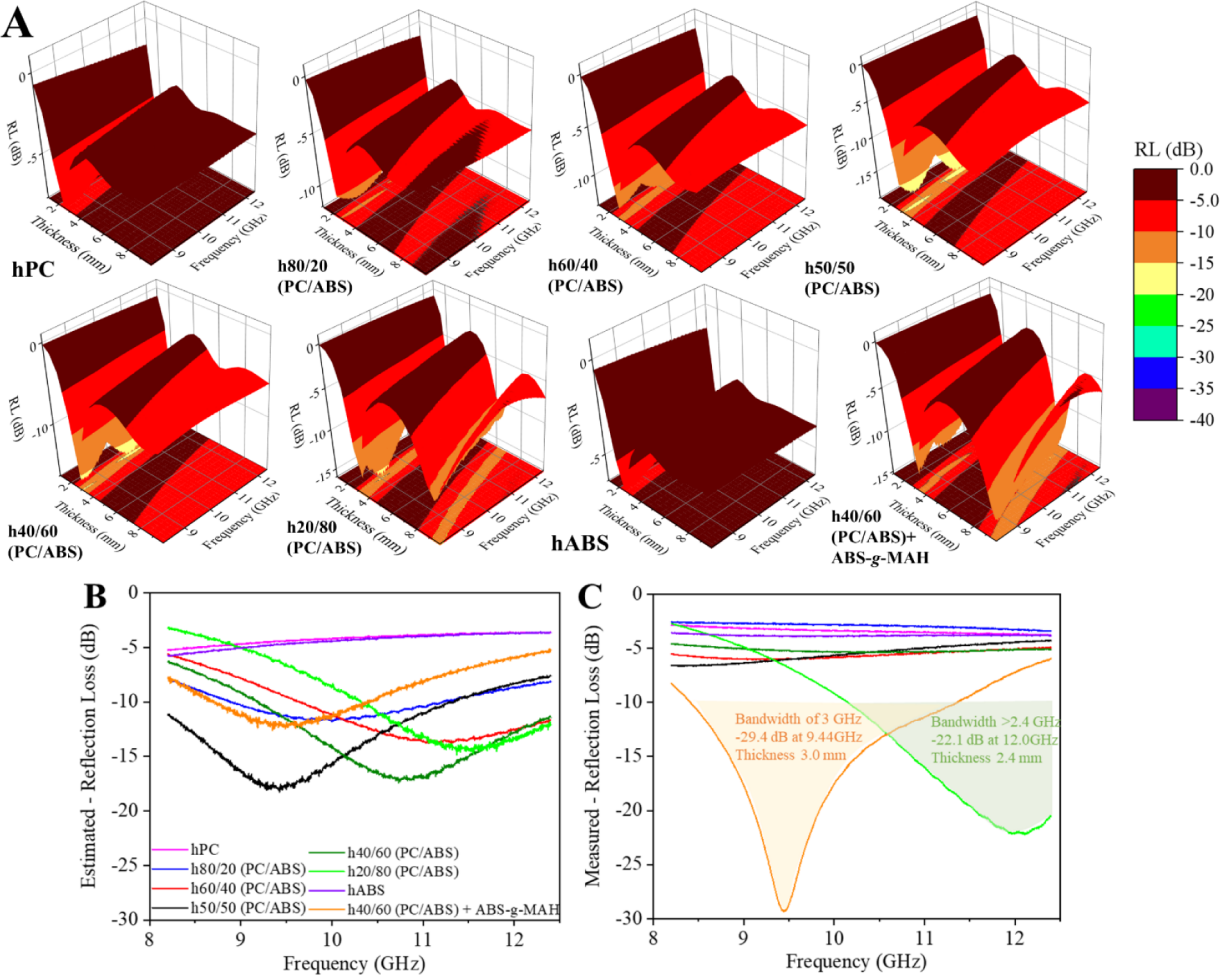
(A) 3D—Mathematically estimated results of the Reflection Loss (RL) behavior from different thicknesses of the PC/ABS blend-based hybrid nanocomposites with different blend ratios for the X-band. Comparison of the estimated (B) and measured (C) RL behavior of the nanocomposites.

From the complex electromagnetic properties, it is possible to mathematically estimate the RL response for different material thicknesses, which is crucial for materials used in microwave absorbers (MAMs).^1,3,11,36^Figure 8A shows the mathematically estimated results of the Reflection Loss (RL) behavior of the PC/ABS blend-based nanocomposites. Unlike common shielding materials, which primarily work by reflecting electromagnetic waves, MAMs are designed to absorb the incident electromagnetic wave energy within a specific frequency range, preventing the signal from being reflected back to the source. According to RL simulations, PC/ABS blend-based nanocomposites demonstrated superior RL behavior compared to single-polymer-based nanocomposites in the X-band frequency range (Figures 8A,B).

The estimated RL behavior was validated by preparing samples with the optimal thickness and measuring their RL performance and these results are shown in Figure 8C. Some compositions did not exhibit the expected RL response, likely due to deviations in absorption bands from the predicted frequency range. However, the RL performance was confirmed for h20/80 (PC/ABS) and h40/60 (PC/ABS) + ABS-*g*-MAH, both of which achieved RL values below −20 dB with bandwidths of over 2.4 and 3.0 GHz, respectively—responses required for commercial applications. Notably, the sparse networks that were less effective in transmission analyses proved highly efficient in generating RL responses. These networks allowed most of the energy to penetrate and be attenuated within the material while reflecting significantly less electromagnetic energy.

A similar result was observed in our previous study, where a single-polymer matrix hybrid nanocomposite, adjusted for higher shear processing, shifted from high shielding behavior to microwave absorption behavior.^11^ Building on this, the results achieved in this study suggest that tuning the polymer blend morphology with a preferential distribution of a single filler can enhance microwave absorption response. Such morphological adjustments could be achieved by altering the blend ratio or incorporating a compatibilizer agent to reduce the size of the second phase of the blend.

### 3.5 Electromagnetic Behavior of the Hybrids on Broadband

Various techniques can be employed to evaluate the electromagnetic behavior of materials, focusing on obtaining their complex electromagnetic properties,^56,57^ as well as their shielding and reflection loss behaviors. Among these techniques, transmission line methods are some of the most commonly used for developing and testing new materials.^3^ These methods can be performed in rectangular waveguides and coaxial lines.

Rectangular waveguides are widely utilized due to their accuracy and repeatability, as they allow the application of higher power and better control over electromagnetic wave propagation modes.^57^ However, to ensure the propagation of the dominant TE_10_ mode, the size of the rectangular waveguide must be controlled within a specific frequency range. This imposes limitations such as a narrow frequency range and large sample size requirements for lower frequencies, as the dimensions of the waveguide dictate the frequency range and sample size.^56^ In contrast, coaxial-line analyses operate in TEM mode and offer a broader frequency range with the same sample size. However, preparing the sample can be more challenging,^58^ and the method is more susceptible to the propagation of undesirable modes in the case of sample defects.^56,57^

**Figure 9:**
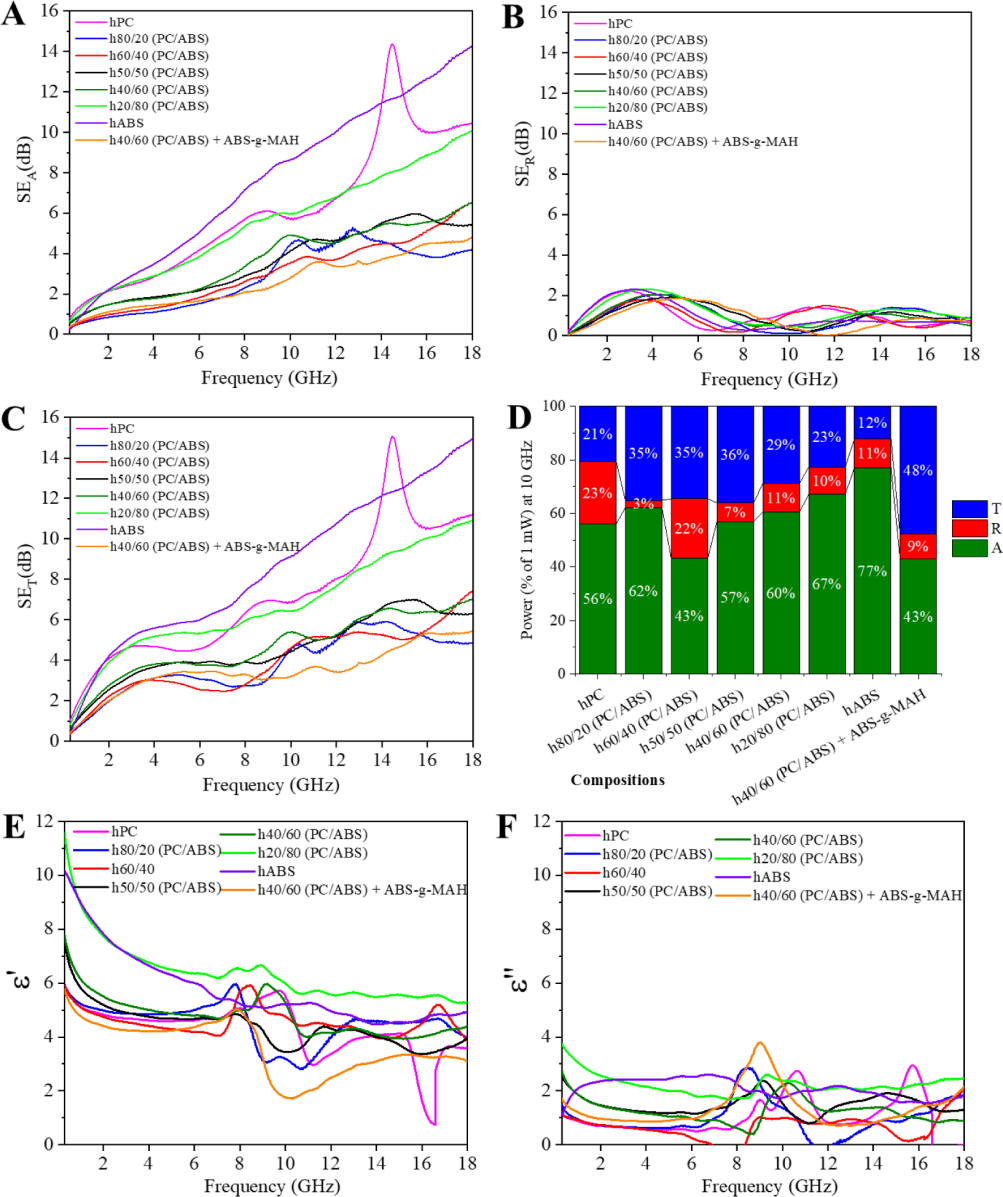
Broadband electromagnetic shielding behavior measured in a coaxial transmission line for the PC/ABS blend-based hybrid nanocomposites with different blend ratios: (A) Attenuation by absorption -SE_A_, (B) Attenuation by Reflection -SE_R_, (C) Total Attenuation -SE_T_, (D) Power coefficients distribution at 10 GHz, (E) real permittivity - ε’, and (F) imaginary permittivity - ε″.

The nanocomposites were also analyzed using a coaxial-line setup to assess their broadband response over a frequency range of 300 MHz to 18 GHz, as shown in Figure 9A. The broadband results exhibited similar trends to those observed in the X-band analysis, with single-polymer matrix nanocomposites demonstrating higher attenuation. Differences in attenuation across compositions were primarily attributed to variations in the shielding by absorption component. However, the attenuation and permittivity values from the broadband analysis were significantly lower compared to those from the rectangular waveguide analysis, potentially due to gaps between the sample and the airline^57^ or differences in sample preparation techniques (Figure S2).

Notably, some variations were observed compared to the X-band results. For instance, hABS exhibited higher attenuation than hPC, and the h20/80 (PC/ABS) composition showed the highest attenuation among the PC/ABS blend-based nanocomposites. Furthermore, across the entire frequency range, attenuation in the low-frequency range (300 MHz to approximately 5 GHz) was dominated by reflection, while attenuation at higher frequencies was primarily driven by absorption. This trend is evident in the power coefficients at 10 GHz, where most of the attenuation is associated with the absorption coefficient (Figure 9D).

For the compositions exhibiting better attenuation (hABS, hPC, and h20/80 (PC/ABS)), a frequency-dependent increase in attenuation, both by absorption and total attenuation, was observed. This behavior, unique to the broadband analysis, can be explained by the attenuation coefficient (α),^59^ which is directly proportional to frequency (*f*), as expressed by eq 9:^59^
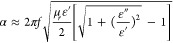
9

This equation assumes that the material is nonmagnetic, as is the case with the studied nanocomposites, and reflects the relationship between the internal attenuation of the material and the applied frequency.^59^

**Figure 10:**
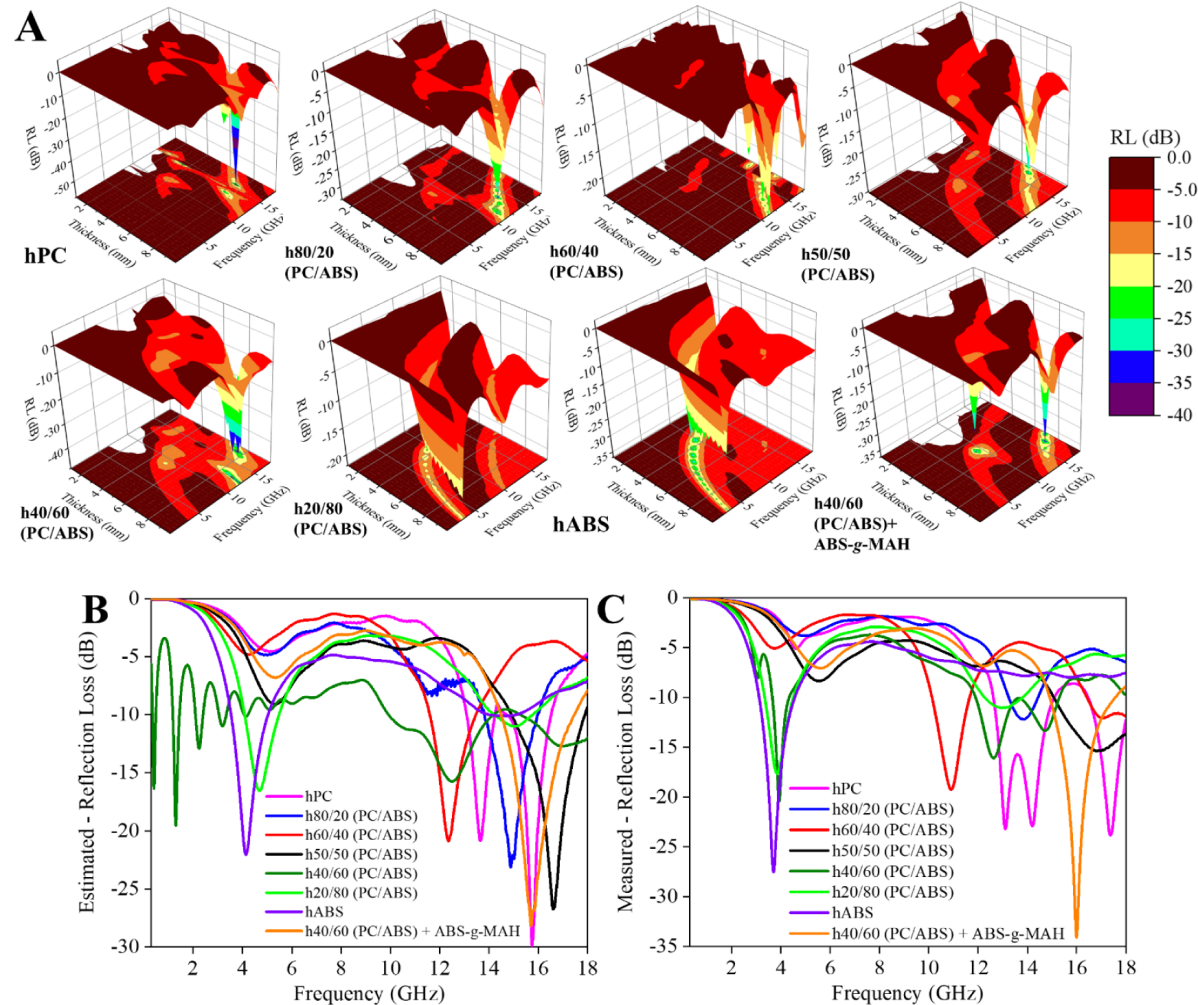
(A) 3D—Mathematically estimated results of the Reflection Loss (RL) behavior from different thicknesses of the PC/ABS blend-based hybrid nanocomposites with different blend ratios for the broadband analysis. Comparison of the estimated (B) and measured (C) RL behavior of the nanocomposites with 7.0 mm samples.

The RL response for the PC/ABS blend-based hybrid nanocomposites was both mathematically estimated and experimentally measured, and the results are shown in Figure 10. The estimated RL response indicated interesting behavior across all nanocomposites, with most exhibiting RL peaks at different frequencies. This variation in frequency response is particularly noteworthy because it suggests that by adjusting the blend ratio or adding a compatibilizer agent it is possible to control the distribution of nanofillers while maintaining content levels. This provides a potential solution for tailoring microwave absorbing materials (MAMs) to specific frequencies when thickness is a constraint. The RL behavior observed in the broadband analysis aligns with the X-band results and it is observed across all nanocomposites in the broader frequency range.

In contrast to the X-band analysis, the compositions hABS and h20/80 (PC/ABS), which exhibited the highest attenuation values, also demonstrated the most promising RL responses. Notably, hABS achieved an RL below −25 dB, which can be tuned to various frequencies by adjusting the material’s thickness. This tunability is a key factor in selecting MAMs for real-world applications, where the performance at specific frequency is often critical.

The shift in behavior from the X-band observations does not present a contradiction. In the broadband analysis, the nanocomposites exhibited a more absorption-dominated response with lower total attenuation values. The experimentally measured RL in the broadband analysis closely matched the calculated estimates for most compositions, though some deviations were noted in terms of frequency or magnitude. Nevertheless, several interesting RL responses were observed, which are summarized in Table 2, detailing key MAM characteristics such as minimum RL, center frequency, and bandwidth.

**Table 2:** RL Responses Lower than −10 dB Were Measured for the PC/ABS Blend-Based Hybrid Nanocomposites with Different Blend Ratios with Samples with 7.0 mm Thickness

	Main peak			Secondary peaks		
Samples	Minimum RL (dB)	Center Frequency (GHz)	Bandwidth (GHz)	Minimum RL (dB)	Center Frequency (GHz)	Bandwidth (GHz)
hPC	–23.8	17.4	>1.53	–23.0/ -22.6	13.1 /14.3	2.45
h80/20 (PC/ABS)	–12.2	13.9	1.19	--	--	--
h60/40 (PC/ABS)	–19.2	10.9	1.55	–12.0	17.0	>1.6
h50/50 (PC/ABS)	–15.4	16.8	2.7	--	--	--
h40/60 (PC/ABS)	–20.4	3.93	0.72	–16.0/ -13.2	12.6/ 14.7	3.38
h20/80 (PC/ABS)	–17.6	3.86	1.15	–11.0	13.1	1.49
hABS	–27.5	3.7	1.46	--	--	--
h40/60 (PC/ABS) + ABS-*g*-MAH	–34.1	16.0	2.66	--	--	--

### 3.6 Mechanical Behavior of the Hybrid Nanocomposites with Different Blend Ratios

For practical applications, presenting only the absorption properties is insufficient for microwave-absorbing materials (MAMs); mechanical performance must also be considered.^3^ The mechanical behavior of the PC/ABS blend-based hybrid nanocomposites was evaluated through tensile tests and Izod impact strength tests, with the results summarized in Table 3.

**Table 3:** Mechanical Behavior of PC/ABS Blend-Based Hybrid Nanocomposites with Different Blend Ratios^a^

Samples	E (MPa)	UTS (MPa)	Elongation (%)	Impact strength (kJ/m^2^)
**hPC**	1865 ± 92^D^	61 ± 3^A^	4.9 ± 1.0^A^	4.8 ± 0.2^A^
**h**80/20 **(PC/ABS)**	2171 ± 23^BC^	55 ± 4^A^	3.2 ± 0.6^B^	1.9 ± 0.1^BC^
**h**60/40 **(PC/ABS)**	2215 ± 45^BC^	44 ± 5^B^	2.1 ± 0.3^C^	1.6 ± 0.1^C^
**h**50/50 **(PC/ABS)**	2128 ± 140^C^	43 ± 4^B^	2.1 ± 0.4^C^	1.5 ± 0.1^C^
**h**40/60 **(PC/ABS)**	2156 ± 102^C^	37 ± 5^BC^	1.6 ± 0.2^C^	1.6 ± 0.1^C^
**h**20/80 **(PC/ABS)**	2483 ± 57^A^	37 ± 7^BC^	1.5 ± 0.3^C^	2.0 ± 0.1^BC^
**hABS**	2179 ± 65^BC^	41 ± 2^BC^	2.0 ± 0.1^C^	2.9 ± 0.2^B^
**h**40/60 **PC/ABS) + ABS-***g***-MAH**	2345 ± 55^AB^	30 ± 3^C^	1.4 ± 0.1^C^	4.3 ± 0.7^A^

a Indexes represent significantly different groups as determined by posthoc Tukey’s test following ANOVA, with a significance level of 0.05 and p-values <0.001 for all properties. Averages that do not share the same letter (A, B, C, D) are significantly different from each other.

First, it is essential to evaluate and compare the mechanical behavior of the single polymer matrix nanocomposites. The elastic modulus (E) of hABS was observed to be higher than that of hPC, likely due to the inherent characteristics of the matrices. In contrast, an inverse trend was found for the ultimate tensile strength (UTS), with hPC showing values around 61 MPa, compared to 41 MPa for hABS. This aligns with the classification of PC as an engineering polymer known for its high tensile strength.^7^

For compositions with a dispersed second phase morphology, such as h80/20 (PC/ABS) and h20/80 (PC/ABS), the addition of 20 wt % of the second phase had a positive effect on the elastic modulus, resulting in a significant increase compared to the single-polymer nanocomposites. In the case of other polymer blend nanocomposites, the effect appeared more additive, as observed in the UTS values across the PC/ABS blend-based nanocomposites.

All nanocomposites exhibited low elongation and impact strength, which can be attributed to the presence of stiff particles acting as stress concentrators or inducing microcracks within the polymer matrix.^7^ However, the addition of ABS-*g*-MAH as a compatibilizer significantly increased both the elastic modulus and impact strength, likely due to the morphological refinement it induced.

As mentioned earlier in this section, the selection of materials for MAMs applications should be approached from a multiproperty perspective, rather than focusing solely on the electromagnetic response.^3^ Considering that the materials demonstrate processability via industrial-like melt mixing methods, which accommodate high-volume demand, the mechanical properties of the nanocomposites presented here offer valuable insights. These properties can help evaluate whether the nanocomposites meet the requirements for specific MAMs projects, in comparison to currently used materials.

## 4 Conclusions

PC/ABS blend-based hybrid nanocomposites, with the simultaneous additions of MWCNT and nanographite for microwave absorption materials applications, were successfully prepared using industrial-like melt-mixing strategies. The nanocomposites were prepared with varying blend ratios (100/0, 80/20, 60/40, 50/50, 40/60, 20/80, and 0/100) in PC/ABS matrices. Morphological and rheological analyses revealed that nanocomposites with blend ratios of 80/20 and 20/80 exhibited a dispersed matrix morphology, while the other ratios studied resulted in a cocontinuous morphology. All nanocomposites demonstrated high electrical conductivity (10^-3^ to 10^-2^ S/cm), largely due to the uniform filler content (2 wt % of MWCNT and 2 wt % of nanographite). The influence of morphological variations and the addition of a compatibilizer agent (ABS-*g*-MAH) significantly impacted the electromagnetic properties, which were evaluated using both rectangular waveguide (X-band) and coaxial airline (broadband) techniques. In the X-band analysis, the nanocomposite based on the 40/60 (PC/ABS) + ABS-*g*-MAH showed promising Reflection Loss (RL) values, with a peak of −29.4 dB at 9.44 GHz and a bandwidth of 3 GHz for a 3.0 mm thick sample, attributed to the refined morphology and nanofiller distribution. The broadband analysis further demonstrated that all nanocomposites achieved RL values below −10 dB across different frequencies, indicating that tuning the matrix morphology is a promising approach for optimizing microwave absorption performance.

## Supporting Information Available

Complex permeability components: A and B (X-band); C and D (broadband); X-band range on the broadband analysis results (PDF)
