# Supplementary material for: Tuning the Morphology of Immiscible Polymer Blend-Based Hybrid Nanocomposite for Improving Microwave Absorption Response
Source: ACS Polym Au. 2024 Nov 28;5(1):10–25. doi: 10.1021/acspolymersau.4c00087 (PMC11826488; doi:10.1021/acspolymersau.4c00087)
Supplement: Supplementary file 1 — lg4c00087_si_001.pdf [file lg4c00087_si_001.pdf]

Supporting Information

Tuning the morphology of immiscible Polymer blend-based Hybrid nanocomposite for improving microwave absorption response

Authors: Erick Gabriel Ribeiro dos Anjos[1,2,3,4], Tayra Rodrigues Brazil[3], Mirabel Cerqueira Rezende[3], Juliano Marini[1,2], Uttandaraman Sundararaj[4], Luiz Antonio Pessan[1,2], and Fabio Roberto Passador[3].

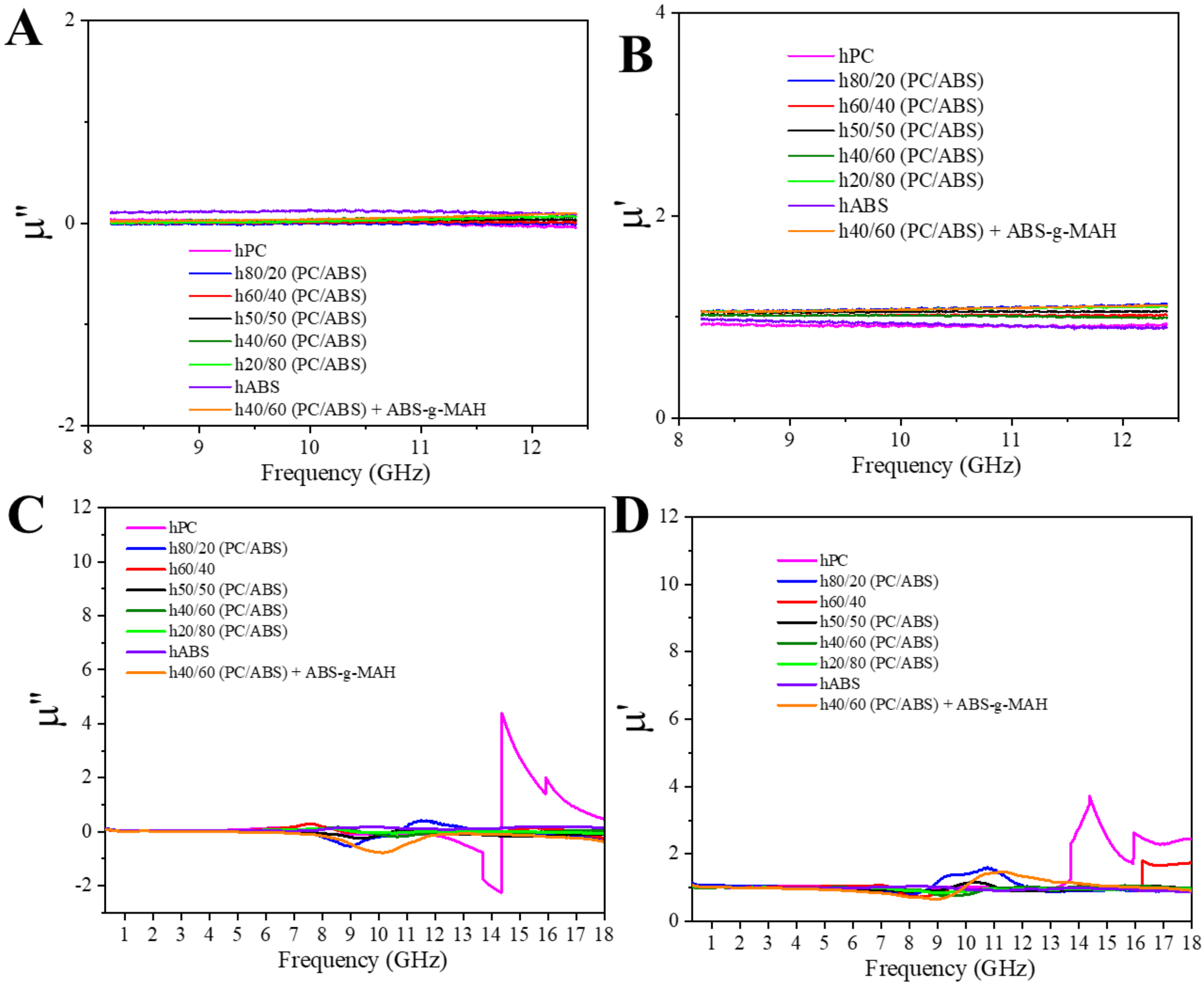

Figure S1. Complex Permeability components: A and B (X-band); C and D (Broadband)

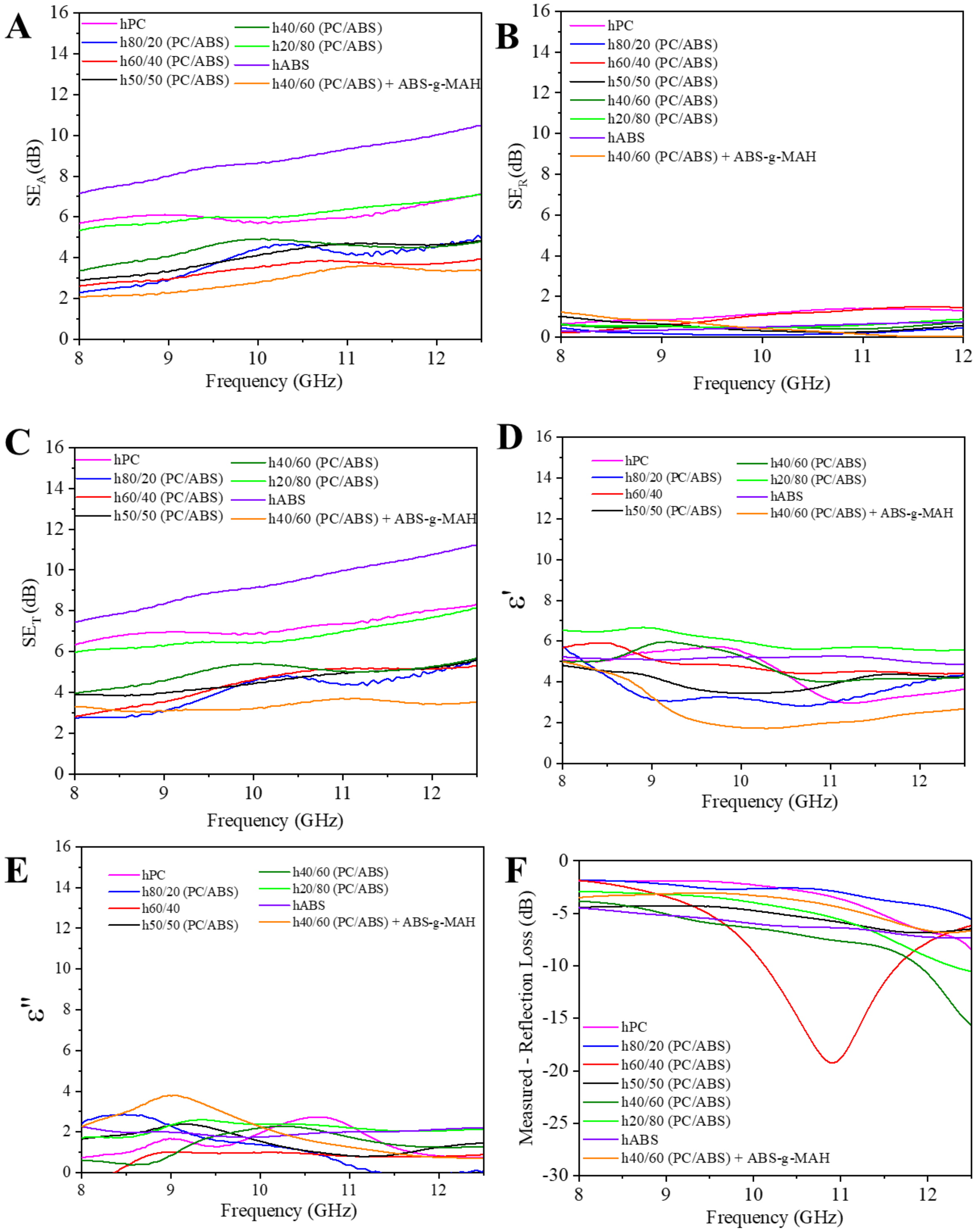

Figure S2. X-band range on the broadband analysis results.
